# Dengue in Java, Indonesia: Relevance of Mosquito Indices as Risk Predictors

**DOI:** 10.1371/journal.pntd.0004500

**Published:** 2016-03-11

**Authors:** Siwi P. M. Wijayanti, Sunaryo Sunaryo, Suprihatin Suprihatin, Melanie McFarlane, Stephanie M. Rainey, Isabelle Dietrich, Esther Schnettler, Roman Biek, Alain Kohl

**Affiliations:** 1 MRC-University of Glasgow Centre for Virus Research, Glasgow, Scotland, United Kingdom; 2 Public Health Department, Faculty of Health Sciences, University of Jenderal Soedirman, Purwokerto, Indonesia; 3 Vector Borne Disease Research and Development Unit, Banjarnegara, Indonesia; 4 Parasitology Department, University of Gadjah Mada, Yogyakarta, Indonesia; 5 Boyd Orr Centre for Population and Ecosystem Health, Institute of Biodiversity, Animal Health and Comparative Medicine, College of Medical Veterinary and Life Sciences, University of Glasgow, Glasgow, United Kingdom; United States Army Medical Research Institute of Infectious Diseases, UNITED STATES

## Abstract

**Background:**

No vaccine is currently available for dengue virus (DENV), therefore control programmes usually focus on managing mosquito vector populations. Entomological surveys provide the most common means of characterising vector populations and predicting the risk of local dengue virus transmission. Despite Indonesia being a country strongly affected by DENV, only limited information is available on the local factors affecting DENV transmission and the suitability of available survey methods for assessing risk.

**Methodology/principal findings:**

We conducted entomological surveys in the Banyumas Regency (Central Java) where dengue cases occur on an annual basis. Four villages were sampled during the dry and rainy seasons: two villages where dengue was endemic, one where dengue cases occurred sporadically and one which was dengue-free. In addition to data for conventional larvae indices, we collected data on pupae indices, and collected adult mosquitoes for species identification in order to determine mosquito species composition and population density. Traditionally used larval indices (House indices, Container indices and Breteau indices) were found to be inadequate as indicators for DENV transmission risk. In contrast, species composition of adult mosquitoes revealed that competent vector species were dominant in dengue endemic and sporadic villages.

**Conclusions/significance:**

Our data suggested that the utility of traditional larvae indices, which continue to be used in many dengue endemic countries, should be re-evaluated locally. The results highlight the need for validation of risk indicators and control strategies across DENV affected areas here and perhaps elsewhere in SE Asia.

## Author Summary

Geographically and economically, Indonesia is one of the most prominent countries in SE Asia, yet many of its endemic infectious diseases are poorly managed, controlled and understood. This includes dengue virus (DENV), which can result in serious human disease and is transmitted by mosquitoes. Dengue risk assessment is a key factor in managing the impact of infection on public health, and this often relies on assessing the presence and nature of mosquitoes through a number of indices associated with the occurrence of larvae and the location/availability of breeding containers. Here we assessed traditionally used indices in combination with other indicators including pupae indices, and the presence of adult mosquitoes in areas with different dengue status: endemic, sporadic or free. Our data suggested that traditional indices were poor indicators of reported local DENV transmission. This has important consequences for design and focus of risk management strategies and efforts to control DENV locally as well as elsewhere in the region.

## Introduction

Dengue virus (DENV) is considered to be the most important arbovirus world wide, with a heavy disease burden in humans [1]. It is transmitted mainly by *Aedes aegypti* mosquitoes, but *Ae*. *albopictus* can also act as a vector [2–5]. Dengue is endemic in many countries around the world, especially in the tropics; moreover the number of endemic areas is increasing [6]. DENV belongs to the genus *Flavivirus* in the family *Flaviviridae* and consists of four antigenically distinct and medically relevant serotypes, with a possible fifth recently described (DENV1, 2, 3, 4 and 5)[1,6–9]. The clinical spectrum of DENV infection can vary from asymptomatic to more severe forms such as dengue hemorrhagic fever (DHF) and dengue shock syndrome (DSS) [8]. DENV is transmitted to humans following mosquito bite (horizontal transmission), and mosquitoes can become infected by ingestion of a DENV-containing blood meal [2]. However, DENV can also be maintained via vertical transmission i.e. passed into eggs and subsequently into the next generation of mosquitoes, thereby maintaining outbreaks in human populations. This has already been documented in both *Ae*. *aegypti* and *Ae*. *albopictus* in different countries including in SE Asia [10–13].

Since it was first identified in 1968 in the cities of Jakarta (capital of Indonesia) and Surabaya (East Java), dengue disease has been recognised as an important public health problem in Indonesia. Periodic outbreaks have occurred in Indonesia with an increasing number of cases and severity [14]. DENV incidence in Indonesia has been shown to peak during the rainy season (between the months of October and April) [15]. From 2004 onwards, Indonesia reported the highest number of DENV cases in the region. All four serotypes of DENV have been found to be circulating since and DENV3 infections associated with the most severe disease [16,17]. Despite dengue being a major concern remarkably little is known or done to control this virus in Indonesia, in spite of its size (in surface and population, as the world’s largest island nation but with high levels of poverty) and important economical position in SE Asia and the world [18].

As there are no vaccines or drugs available for DENV, control programmes for DENV transmission are often focused on managing mosquito populations. To determine the nature of mosquito populations, entomological surveys are usually conducted within routine control programmes [19]. For many years, the standard protocol has relied on traditional sampling (*Stegomyia* indices) which is based solely on the presence of larvae [20]. Indicators of DENV vector abundance (mainly *Ae*. *aegypti*) were based on larval surveys of container habitats and the calculation of various indices, namely House Index (HI: percentage of houses infested with larvae or pupae), Breteau Index (BI: number of larvae or pupae positive containers per 100 houses examined) and Container Index (CI: percentage of water-holding containers found to be infested with larvae or pupae) [20]. These indices can facilitate understanding of vector ecology in a given control area, but also serve as useful measures to determine the success of intervention strategies. However, these traditional sampling methods have shortcomings by measuring only the abundance of larvae and not determining species, which therefore may be poor predictors of the abundance of adult vector mosquitoes that are responsible for transmission [20]. Following consideration of these issues, Focks (2003) suggested that pupal/demographic survey methods were developed to replace the more traditional larval indices [20]. The pupae index is based on counting the number of pupae per container and identifying which container types are responsible for the largest number of adult mosquitoes. It assumes the ability to predict the potential of DENV epidemics more accurately than the traditional HI, which does not necessarily correlate with DENV transmission [21,22].

Despite the limitation of traditional sampling methods, many studies have continued to focus on indices from larval stages of the mosquito, e.g. [23–28]. However, the importance of developing improved and locally appropriate entomological surveys in DENV endemic areas is increasingly recognised [20]. In Indonesia, variations in dengue disease reporting make it important to further understand the entomological differences between areas with different DENV risk i.e., in endemic areas (defined here as an area that has regularly reported DENV cases in the three years preceding this study, 2009–2011), sporadic areas (defined here as an area which has had an irregular number of DENV cases reported in the three years preceding this study, 2009–2011) and compare these to dengue free areas (defined here as an area with no reports of dengue disease in the three years preceding this study, 2009–2011). Comparisons of mosquito populations and local habits in these different areas are more likely to indicate the key entomological differences that can inform potential points of intervention, and the validity of the various indices and survey methods.

In this study we applied traditional larvae indices, the pupae index as well as adult mosquito collections and species identification to compare and enhance the validity of entomological survey results in villages with different dengue endemicity in the Banyumas Regency of Java. By comparing these traditional indices to newer indicators with respect to their ability to predict dengue risk, we aimed to better understand the local dengue transmission processes. Overall our data help to fill important gaps in our knowledge of dengue transmission and associated ecology/human behaviour in this area of SE Asia and inform local prevention strategies. Our observations may be relevant beyond the study area by informing entomological surveys elsewhere. By determining the most representative factors to predict/analyse mosquito populations and transmission risk, entomology surveys can be done in an effective and more efficient manner.

## Methods

### Description of the study area

**Fig 1:**
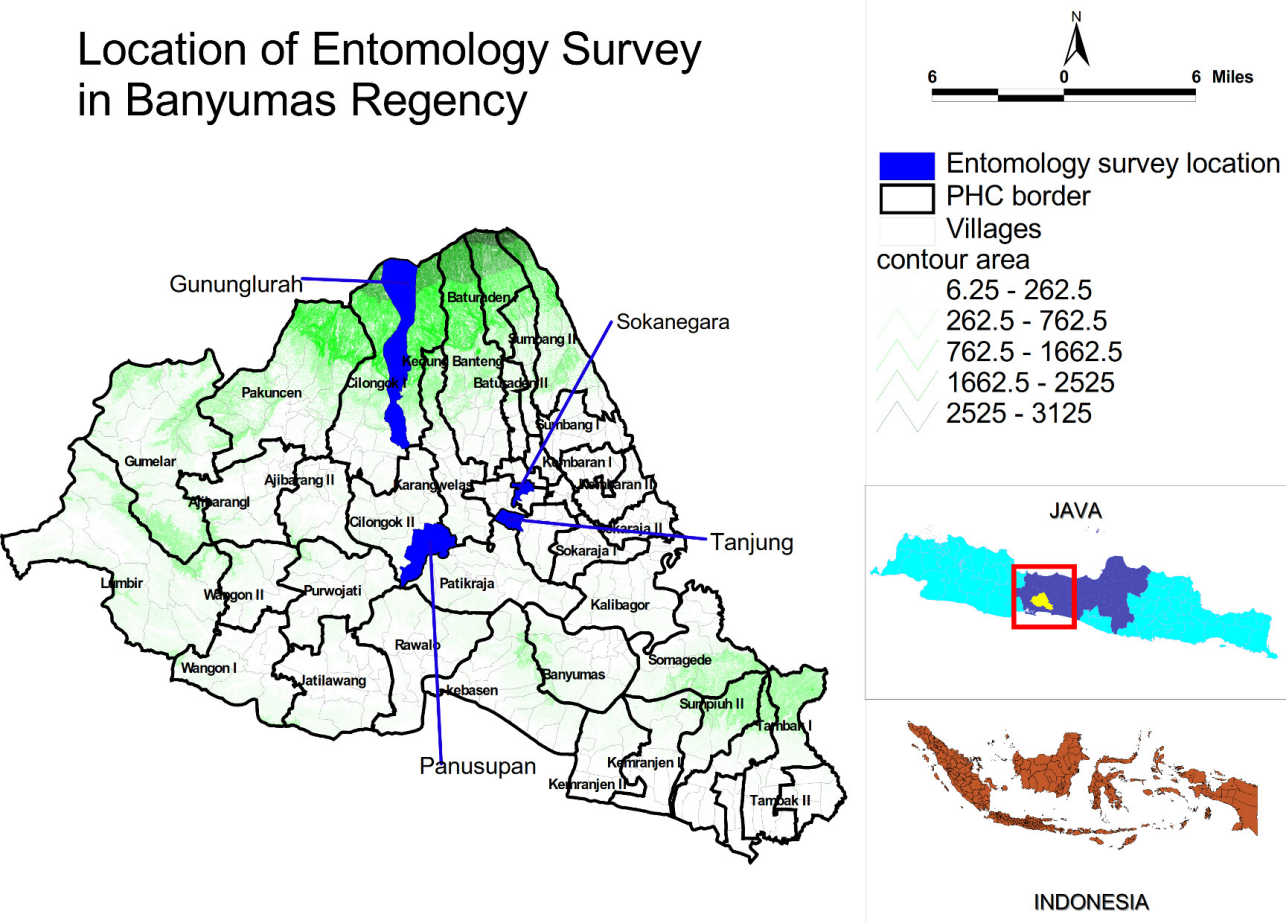
Location of the entomology survey areas in the Banyumas regency. Black areas indicate the locations of the entomology survey: Tanjung, Sokanegara, Panusupan and Gunung Lurah. This map also shows the contours of the area (in meters). Lower right, map of Java with Banyumas regency (in white) and also whole map of Indonesia.

The study site used in this analysis is the Banyumas Regency, located in the southwest of Central Java Province, Indonesia (Fig 1). Coordinates for this location are as follows: 108" 39`17”–109" 27`15” East longitude, and 7" 15`05”–7" 37`10” South latitude. The total area is 132,760 km^2^, with a population of 1.85 Million inhabitants at a male to female ratio of 50:50. Banyumas Regency consists of 27 sub districts, and has 39 community health centres and a total of 331 villages. The environment in Java is characterized by a tropical monsoonal climate, with a dry season lasting approximately 6 months and a heavy monsoon the rest of year. Total annual precipitation averages at 1755 mm (69.1 inches) and there are 2975 hours of sunshine on average per year.

### Household studies and seasonal analysis

In order to determine the differences in mosquito population density between seasons, entomological surveys were carried out twice, once in the dry and once in the rainy season. During the dry season three independent villages were selected on the basis of differing endemicity status and their spread across the region (Fig 1): DENV endemic: Tanjung village, South Purwokerto Community Health Centre and Sokanegara, East Purwokerto Community Health Centre; DENV sporadic: Panusupan village, Cilongok I Community Health Centre; DENV free: Gunung Lurah village, Cilongok II Community Health Centre.

The endemicity status criteria are based on “The Technical Manual Eradication of Dengue Mosquito-borne Diseases, Indonesian Ministry of Health” (1992) [29]. The determination of DENV status was made before the survey began, based on reported dengue cases from the Banyumas Regency Health Office. All suspected DENV cases are reported to the health office, however not all cases are confirmed. It is important to note that all reported cases are severe and require hospitalisation. Therefore it is likely there is under reporting of actual cases across the regency. The dry season survey was conducted from May-June 2012, while the rainy season survey was conducted from January-February 2013. One additional village, Sokanegara village (DENV endemic) was added to the survey in the rainy season. In each village, 100 houses were chosen by simple random sampling for the entomological surveys, resulting in a total of 300 houses being analysed in the dry season and 400 houses in the rainy season. The individual locations of the entomological surveys are shown in Fig 1. An overview of environmental conditions and characteristics of the four villages is shown in Table 1.

**Table 1:** Description of the study sites in Banyumas Regency, Java. Details of the ecology and population of the four study sites are provided.

Study sites		Ecological description							Dengue cases in the years before the survey		
	Urban/rural	Population	Elevation (metres above sea level)	Coverage area (ha)	Rainfall in dry season (Apr-Sept(mm)	Rainfall in rainy season (Oct-March) (mm)	Range of temperature (in °Celsius)	Range of humidity (%)	2009	2010	2011
Tanjung (DENV endemic)	Urban	9696	65	149	796	1858	28–32	73–91	7	13	14
Sokanegara (DENV endemic)	Urban	7987	75	119	731	1705	30–38	57–83	9	17	6
Panusupan (DENV sporadic)	Rural	7627	200	775	885	2065	29–36	70–87	1	2	0
Gunung Lurah (DENV free)	Rural	7120	400–700	878	765	1785	25–34	57–83	0	0	0

### Larval/pupae surveys and adult mosquito collections

Larvae collection was carried out in every container both inside and outside the participating houses. To measure the entomological parameters the House Index (HI: percentage of houses infested with larvae and/or pupae), Container Index (CI: percentage of water-holding containers infested with larvae or pupae), Breteau Index (BI: number of positive containers per 100 houses inspected), Pupae Index (PI: number of pupae per 100 houses inspected) and Free Larvae Index (FLI: the percentage of houses without larvae) were determined [20]. 100 houses is the sample size recommended by the Indonesian Ministry of Health, in “The Technical Manual Eradication of Dengue Mosquito-borne Diseases, Indonesian Ministry of Health” (1992) [29]. The interpretation of transmission risk levels of each village was made based on the larvae index, as described in the WHO document “A review of entomological sampling methods and indicators for dengue vectors” [20]. The survey also included a description of all containers, both artificial and natural in each participant’s house. Identification of the recovered larvae was based on the key identification criteria as described by Stojanovich and Scott, 1965 [30].

Insect collections were carried out using back-pack aspirators to capture adult mosquitoes in resting and flying positions. Areas inside the house where mosquitoes normally rest were focused on. For example, *Ae*. *aegypti* mosquitoes prefer to rest in dark, shielded, humid areas on hanging objects such as clothes and curtains and on walls. Adult mosquito capture was carried out between 8–11 AM for around 20 minutes per house (100 houses per village). Identification of adult mosquitoes was conducted by using key identification criteria as described earlier [30]. Sample sizes of 100 houses were used as recommended.

### Statistical tests

Confidence intervals (C.I.) 95%, t test, chi square test were calculated by using IBM SPSS Statistic 21.

### Ethical statement

Studies conducted here (data collection of mosquito breeding sites, mosquito egg collections) were carried out with ethical approval from the University of Glasgow (Project Number: 2012082) and the Ministry of National Education, Faculty of Medicine Gadjah Mada University, Medical and Health Research Ethics Committee (KE/FK/323/FC). No data involving human participants were collected in this study.

## Results

### Larvae and pupae: survey results inside and outside houses

Following the field surveys conducted during the dry and rainy seasons, the HI and BI indices in the rainy season were found to be higher than in the dry season: the average HI and BI in all villages in the rainy season were 24 and 31, respectively, higher than in the dry season (15 and 18, respectively). On the other hand, the CI was lower in the rainy season (Table 2) probably because more containers were found in the rainy seasons in all villages. Thus compared to the dry season, more mosquito larvae were found during the rainy season. Panusupan (DENV sporadic) showed the lowest free larvae indices (FLI), and was classed as a high risk level of DENV transmission compared to other villages using this index.

**Table 2:** Risk level of dengue transmission based on larvae indices in the 4 study areas in the dry and rainy seasons. The following table summarizes House index (HI), Breteau Index (BI), Container Index (CI) and Free Larvae Index (FLI). Risk level determination according to AHA Brown [20]; C.I.: confidence interval.

Village	DENV status	Larvae index, dry season					Larvae Index, rainy season				
		HI (95% C.I.)	BI (95% C.I.)	CI (95% C.I.)	FLI (95%C.I.)	Risk Level	HI (95%C.I.)	BI (95% C.I.)	CI (95% C.I.)	FLI (95% C.I.)	Risk Level
Tanjung	Endemic	16 (8–23)	16 (8–23)	10 (5–14)	84 (76–91)	Medium	18 (10–26)	18 (10–26)	4 (2–5)	82(74–90)	Medium
Sokanegara	Endemic	-	-	-	-	-	18 (10–26)	18 (10–26)	5 (2–6)	82 (74–90)	Medium
Panusupan	Sporadic	25 (16–33)	35 (25–44)	22 (15–28)	75 (66–83)	Medium	44 (34–53)	71 (62–79)	13 (9–15)	56 (46–65)	High
Gunung Lurah	free	3 (0–6)	3 (0–6)	3 (0–5)	97 (93–100)	Low	16 (8–23)	19 (11–26)	3 (1–4)	84 (76–91)	Medium
Average		15	18	11	85		24	31	6	76	

To determine whether larvae density correlated with number of DENV cases occurring after the survey, updated information on the number of dengue cases from the Banyumas Regency Health Office was obtained (Table 3).

**Table 3:** Mosquito larvae density in dry and rainy seasons, and the number of dengue cases in 2012 and 2013. Dengue case numbers following the surveys were obtained from Banyumas Health Officer’s Report.

Village	Endemicity status	Mosquito larvae density, dry season (May-June 2012)	Mosquito larvae density, rainy season (Jan-Feb 2013)	Dengue cases, 2012 (July-December)	Dengue cases, 2013 (March-December)
Tanjung	Endemic	Medium	Medium	2	15
Sokanegara	Endemic	-	Medium	13	9
Panusupan	Sporadic	Medium	High	0	0
Gunung Lurah	Free area*	Low	Medium	0	1

* Despite one case, classed as free based on years preceding these surveys (see Table 1). Larval densities were measured in accordance to Focks et al. [20].

**Fig 2:**
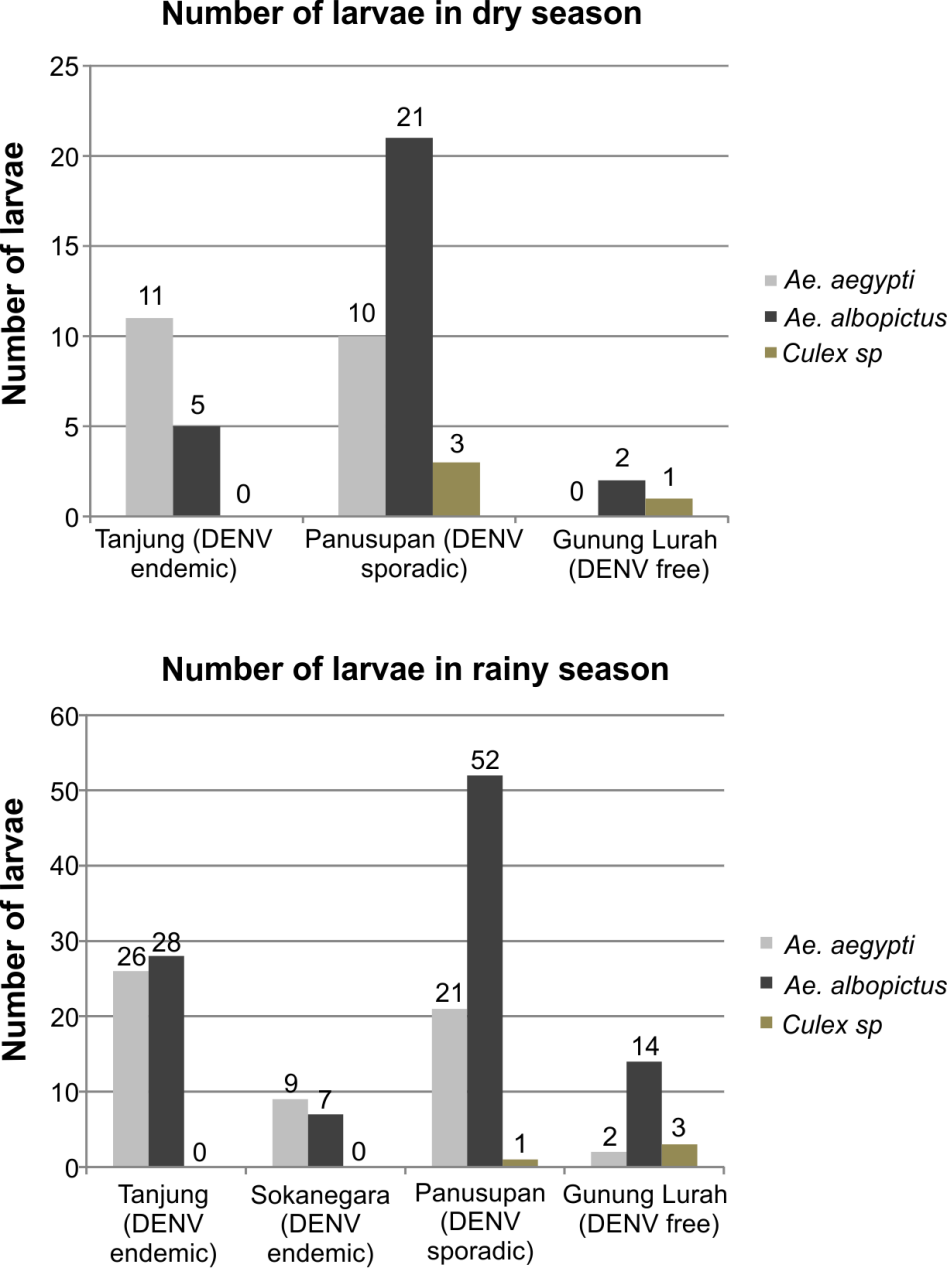
Number of mosquito species within the larvae identified in each of the four villages in dry and rainy season. Each bar represents the mosquito species as indicated in the legend.

Based on the report, Tanjung and Sokanegara (DENV endemic) which were classified as medium risk level according to the indices above, continued to have more dengue cases in 2012 and 2013 compared to the sporadic and free area. Meanwhile, Panusupan (DENV sporadic, but classed as high risk) reported no dengue cases in 2012 and 2013 and Gunung Lurah (reported as DENV free before 2012 and with a low or medium risk depending on index used) reported one dengue case in 2013. In addition to calculating the various indices as outlined above, species identification of the collected larvae was performed (Fig 2).

Numbers of larvae in all villages were higher in the rainy season than in the dry season. *Ae*. *aegypti* and *Ae*. *albopictus* were the dominant species in Tanjung, Sokanegara (DENV endemic) and Panusupan (DENV sporadic). *Culex sp*. were identified in low numbers only in Panusupan (DENV sporadic) and Gunung Lurah (DENV free). Next pupae were assessed in each village and the Pupae Indices (PI) used in order to improve the entomology survey. Details of PI (house and container pupae indices) are indicated in Table 4.

**Table 4:** Pupae indices for the four villages included in this study.

Name of Villages	status	CPI (%)(95% C.I.)		HPI (%)(95% C.I.)		Pupae/person		Pupae/house		Pupae/container	
		dry	rainy	dry	rainy	dry	rainy	dry	rainy	dry	rainy
Tanjung	endemic	4 (0–8)	4 (0–8)	5 (0.7–9)	9(3–15)	0.02	0.061	0.09	0.26	0.05	0.05
Sokanegara	endemic	-	5 (1–9)	-	11 (5–17)	-	0.04	-	0.21	-	0.05
Panusupan	sporadic	6 (1–11)	1 (0–3)	7 (2–12)	5 (1–9)	0.03	0.02	0.11	0.11	0.07	0.01
Gunung Lurah	free	3 (0–6)	1 (0–3)	2 (0–5)	2 (0–5)	0.01	0.007	0.04	0.03	0.03	0.005

*CPI: Container Pupae Index; HPI: House Pupae Index; C.I.: confidence interval.

The DENV endemic and sporadic areas had higher CPI and HPI than the DENV free area (Gunung Lurah) (X^2^ = 6.60, df = 1, p-value = 0.01). This indicated that in the endemic/sporadic areas, mosquitoes tend to have a more conducive environment to survive from eggs to become pupae, and environments with greater survival of mosquitoes to the pupal stage correlated to a higher number of reported dengue cases in endemic areas (Table 3) compared to sporadic and free areas; the high CPI and HPI did not however, correlate with the zero reported cases in Panusupan in the months after the survey. According to Focks (2003) the threshold of dengue transmission is when the pupae/person index (Table 4) ranged between 0.5–1.5 with an optimum air temperature 28°C [20]. Containers found in each house were recorded in order to determine what the dominant mosquito breeding containers were in the various study areas. The main finding of the container survey was that more artificial containers were found in the four villages surveyed compared to natural containers (paired t-test, mean 195, SD 310, p = 0.003) (summarized in Table 5). We found more natural containers in sporadic and free areas (Panusupan and Gunung Lurah) compared to endemic areas (Sokanegara and Tanjung), although there was no significant difference (p = 0.5) (Table 5). Endemic areas (Tanjung and Sokanegara) are more urbanised (less vegetation, and more densely populated), as described in Table 1. Buckets, water storage containers and traditional bath-tubs were found to be the dominant breeding containers observed in all four villages. In fact, discarded tyres were the containers which had the highest proportion of infestation (53%), this finding is also consistent with other studies [31,32]. Moreover, other artificial containers such as aquariums, water dispensers and flower pots also showed high infestation rates.

**Table 5:** Proportion of water-holding containers infested with larvae and/or pupae in the four villages of the study area. Both rainy and dry seasons are taken into account. Containers are described by type, as indicated.

Type of water-holding container	Container observed									Container positive with larvae								Total container positive with larvae	Total container without larvae	Proportion infested (%)
	Tanjung		Sokanegara		Panusupan		Gunung lurah		Total observed	Tanjung		Sokanegara		Panusupan		Gunung Lurah				
	dry	rainy	dry	rainy	dry	rainy	dry	rainy		dry	rainy	dry	rainy	dry	rainy	dry	rainy			
Traditional bath tub	75	75		88	63	63	48	48	460	3	8		11	4	12	1	0	39	421	8
Buckets	55	198		152	73	344	62	212	1096	6	5		2	17	24	1	7	62	1034	6
Dispenser	8	16		16	0	8	0	1	49	2	2		0	0	3	0	1	8	41	16
Leaf Midrib	0	10		1	1	13	1	49	75	0	0		0	0	2	0	5	7	68	9
Used bottles	0	57		61	1	14	2	105	240	0	0		0	0	5	0	3	8	232	3
Refrigerator	8	10		17	0	3	0	0	38	1	0		0	0	1	0	0	2	36	5
Flower pot	7	11		1	18	6	3	0	46	1	0		0	9	1	1	0	12	34	26
Water storage/container	10	57		47	2	91	4	130	341	3	2		1	4	16	0	2	28	313	8
Aquarium	3	5		4	0	4	0	0	16	0	0		1	0	1	0	0	2	14	13
Discarded Tires	0	3		4	1	4	0	7	19	0	0		3	1	5	0	1	10	9	53
Drum	0	40		0	0	0	1	0	41	0	1		0	0	1	0	0	2	39	5
coconut shells	0	0		0	1	7	0	0	8	0	0		0	0	0	0	0	0	8	0
	**166**	**482**	** **	**391**	**160**	**557**	**121**	**552**	**2429**	**16**	**18**	** **	**18**	**35**	**71**	**3**	**19**	**180**	**2249**	** 7**

### Adult mosquito field collections: larval indices did not always correspond to abundance of adult vectors

**Fig 3:**
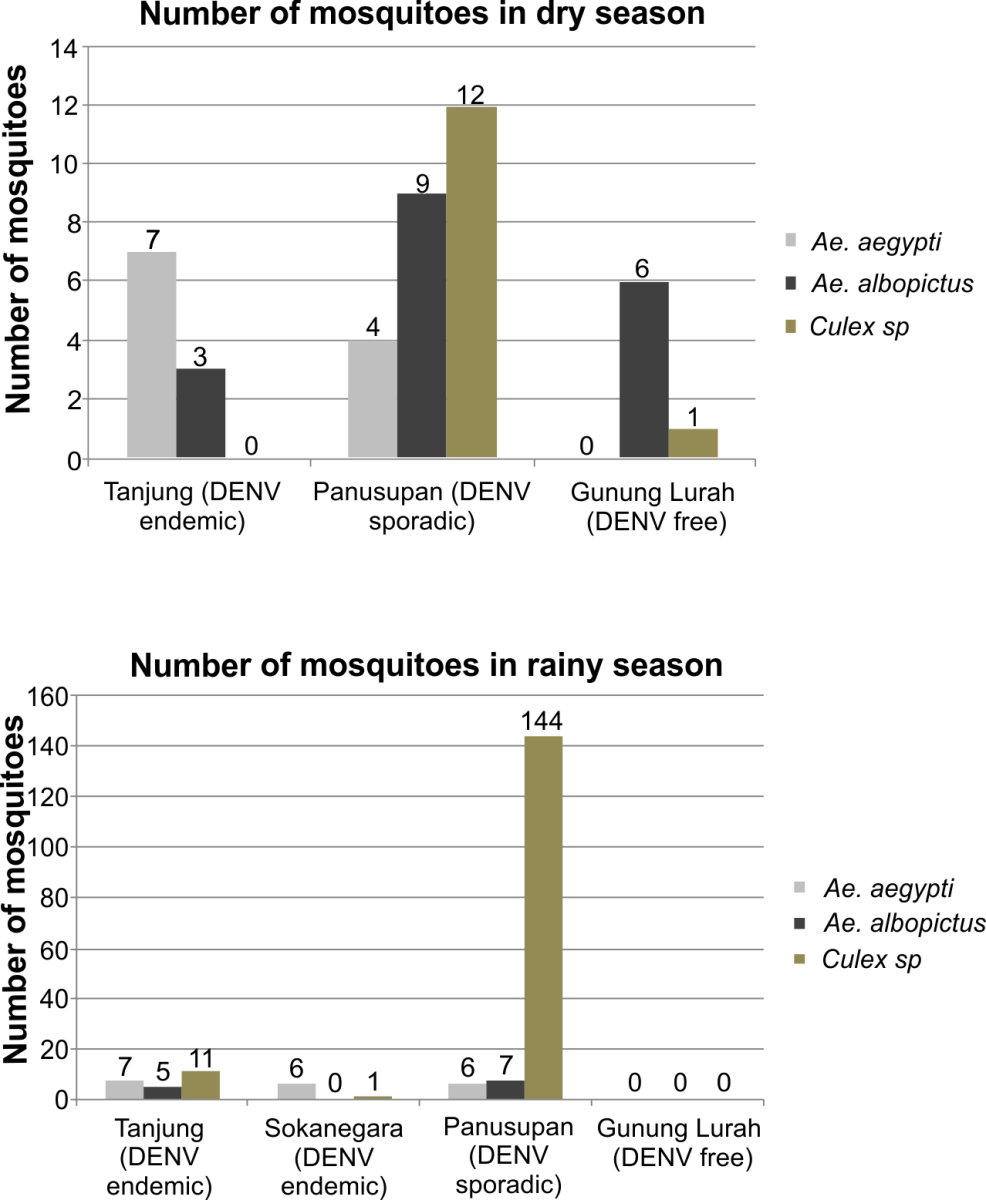
Number of adult mosquito species collected in each of the villages studied in the dry and rainy seasons. Each bar represents the mosquito species indicated as in the legend. Dengue endemicity status is also indicated.

Measuring adult mosquito numbers is considered to be the most representative quantitative estimate to obtain information about mosquito abundance, as immature stages need to go through several developmental stages to become adult mosquitoes before they can transmit DENV [20]. After identification of the mosquito species in the dry and rainy seasons, the numbers of each species in each area are shown in Fig 3.

The dominant adult mosquito species captured (both seasons combined) during the survey in Tanjung (where *Culex sp*. were dominant only in the rainy season) and Sokanegara (DENV endemic) were *Ae*. *aegypti*; in Panusupan (DENV sporadic), non dengue transmitting *Culex sp*. were identified as the dominant species (12 in the dry season and 144 in the rainy season); although comparable numbers (to endemic areas) of *Ae*. *aegypti* were identified. Moreover the combined numbers of *Ae*. *aegypti* and *Ae*. *albopictus* were higher in Panusupan than in the dengue endemic areas. However, because of its feeding preference, the role of *Ae*. *albopictus* has been called into question [3]. This adult collection result is in contrast with the larvae identification, where *Ae*. *albopictus* was found to be the dominant species in Panusupan and this is likely due to breeding behavior, as *Ae*. *albopictus* (and *Ae*. *aegypti*) species frequently breed in containers around housing while culicine mosquitoes use different types of habitats. In Gunung Lurah (DENV free), we captured very few adult mosquitoes, and *Ae*. *albopictus* was the dominant species collected while *Ae*. *aegypti* was completely absent.

## Discussion

Many DENV-endemic countries such as Indonesia, Malaysia and Thailand use entomological surveys as a routine method recommended by WHO to record mosquito populations [19]. Information on mosquito density can then be used in mosquito control efforts and in prevention of DENV transmission [33,34]. Areas with high mosquito populations have usually been treated with larvicides such as organophosphates or temephos in an attempt to prevent outbreaks of DENV. In Indonesia, a Ministry of Health programme encourages community participation in carrying out routine entomology surveys in their homes [35]. Some villages in Indonesia also have trained village health volunteers (VHV) who regularly conduct entomological surveys. Traditional sampling methods i.e. larvae indices were routinely applied over many years to determine mosquito densities in defined areas and the subsequent risk of DENV transmission. However there can be limitations associated with traditional indices [20–22,36]. To assess the validity and usefulness of these methods, and improve the characterisation of vector populations in our study area, we combined the traditional larvae indices together with the pupae index, species identification and adult mosquito collections.

Our results suggest that traditional larvae indices might not always be an appropriate way of quantifying mosquito populations and dengue transmission risk, as has been previously reported [36]. From the adult mosquito collections (and subsequent species identification), the high larvae indices in Gunung Lurah village (DENV free area; one recent case likely to have been imported from an area where transmission occured) did not support the transmission of DENV as very few adult mosquitoes were captured in this village. Nonetheless it is important to point out that both *Ae*. *albopictus* and *Ae*. *aegypti* were present in DENV-sporadic Panusupan and perhaps differences in vectorial capacity come into play locally. Clearly, our data indicated that larvae density was not always in accordance with the number of DENV cases reported in villages. The pupae survey in this study (Table 4), would suggest that the area of study has a low risk for dengue transmission according to factors previously defined by Focks (2003). The high presence of *Ae*. *albopictus* larvae in the dengue free area also suggests that presence of vectors alone may not predict transmission; *Ae*. *albopictus* in this area may have reduced capacity due to their feeding behaviour etc. or possibly reduced competence for DENV. That very few adult mosquitoes were found in 100 houses in Gunung Lurah might be due to a generally unfavourable environment for mosquitoes. These results suggested that high levels of adult *Ae*. *aegypti* in endemic (and sporadic) areas were a potential indicator of DENV transmission risk. These findings were in agreement with the real numbers of DENV cases which occured in Gunung Lurah; one reported case in 2013. Adult mosquito numbers (and species identification) may be a useful estimate to obtain information on dengue disease risk, at least in this part of Indonesia as immature stages need to go through several developmental stages in order to become adult mosquitoes able to transmit DENV [20,36]. However, these methods require specialist skills [37] and are not easily transferable to local surveillance programmes. Moreover, while our observations suggest that the usefulness of several indices should be questioned at local level, we stress that underreporting of dengue cases needs to be taken into consideration in the discussion of our results. Improved patient data collection and dengue diagnostics need to be developed, implemented and combined with future mosquito surveillance work in the Regency to support entomological surveillance studies whose accuracy relies on such data. Our findings may encourage such efforts and lead to a more in depth re-evaluation of the observations reported here.

Based on the results of this study, mosquito populations in the regency are higher in the rainy season than in the dry season, for example more mosquito larvae and also adult mosquitoes in three villages were found during the rainy season compared to the dry season. This suggests that health officers and the community should focus their efforts on the beginning of the rainy season. Not surprisingly we also found more potential breeding containers in the rainy season with buckets and water storage containers as predominant water sources in all four villages surveyed. Our findings indicate that villagers can minimize the potential breeding sites for mosquitoes by reducing the presence of artificial containers such as traditional bath-tubs and buckets.

It can be assumed that by reducing the number of these containers, DENV incidence could be minimized. The results from adult mosquito captures in the four villages indicated that *Ae*. *aegypti* still preferred urban areas (Tanjung and Sokanegara), although in Panusupan (DENV sporadic, rural), *Ae*. *aegypti* was also observed although the numbers of *Culex sp*. mosquitoe*s* in this village were far higher. *Ae*. *albopictus* is more likely to be found in rural or suburban areas. These observations are also emphasized by our container survey, where we observed that natural containers were found more frequently in rural areas (Panusupan and Gunung Lurah) and *Ae*. *albopictus* is more prevalent than *Ae*. *aegypti*. These findings are in accordance with previous reviews on the differences in distribution and ecology between *Ae*. *aegypti* and *Ae*. *albopictus* which stated that *Ae*. *albopictus* prefers natural containers [38].

Species identification is important but rarely applied in the field, and often only for research purposes. *Culex* sp. mosquitoes were identified as the dominant adult mosquito type in Panusupan village; this is of interest since this species has not been shown to be a vector of DENV. Vazeille and colleagues stated that *Ae*. *aegypti* is the most effective vector for dengue viruses and is highly receptive to oral infection; they also demonstrated that *Cx*. *quinquefasciatus* can be infected by the parenteral route with DENV type 2 but the virus replicated to very low levels, therefore the authors concluded that *Cx*. *quinquefasciatus* should not be considered a biological vector of DENV [39].

A recent study carried out in Taiwan suggested that various vector indices alone were poor DENV outbreak indicators and each country should evaluate its own situation [40]. We agree with this statement, although we emphasize that better diagnostics needs to be implemented as part of any future studies on this subject in Java. The transmission risk by adult mosquitoes can be influenced by a number of factors that affect the extrinsic incubation period (EIP) and arbovirus/ vector interactions. Indeed, virus and vector genetics, but also gut microbiota and host responses are important factors in DENV-vector interactions [41–53]. Moreover, climatic factors such as temperature and humidity come into play. The influence of temperature on EIPs associated with DENV for example, has been analysed, and was shown to be important for EIP duration [54–56]. These, and other risk factors may vary locally, and could also change over time highlighting the importance of local assessments. At least in the case of Banyumas Regency, our findings also suggest that more prevention efforts should be carried out in the beginning of the rainy season to reduce dengue virus transmission, for example by clearing artificial containers. In summary the observations of this study can form the basis of a better understanding of dengue vector ecology in this part of Indonesia.
