# Correction: Dengue in Java, Indonesia: Relevance of Mosquito Indices as Risk Predictors

**DOI:** 10.1371/journal.pntd.0004683

**Published:** 2016-04-26

**Authors:** Siwi P. M. Wijayanti, Sunaryo Sunaryo, Suprihatin Suprihatin, Melanie McFarlane, Stephanie M. Rainey, Isabelle Dietrich, Esther Schnettler, Roman Biek, Alain Kohl

In the Funding section, the grant number from the funder the UK Medical Research Council was omitted. The correct grant number is: MC_UU_12014.
